# Brain evolution by brain pathway duplication

**DOI:** 10.1098/rstb.2015.0056

**Published:** 2015-12-19

**Authors:** Mukta Chakraborty, Erich D. Jarvis

**Affiliations:** 1 Department of Neurobiology, Duke University Medical Center, Durham, NC 27713, USA; 2 Howard Hughes Medical Institute, Chevy Chase, MD 20815, USA

**Keywords:** brain pathway, duplication, parrots, song systems, brain evolution, speech

## Abstract

Understanding the mechanisms of evolution of brain pathways for complex behaviours is still in its infancy. Making further advances requires a deeper understanding of brain homologies, novelties and analogies. It also requires an understanding of how adaptive genetic modifications lead to restructuring of the brain. Recent advances in genomic and molecular biology techniques applied to brain research have provided exciting insights into how complex behaviours are shaped by selection of novel brain pathways and functions of the nervous system. Here, we review and further develop some insights to a new hypothesis on one mechanism that may contribute to nervous system evolution, in particular by brain pathway duplication. Like gene duplication, we propose that whole brain pathways can duplicate and the duplicated pathway diverge to take on new functions. We suggest that one mechanism of brain pathway duplication could be through gene duplication, although other mechanisms are possible. We focus on brain pathways for vocal learning and spoken language in song-learning birds and humans as example systems. This view presents a new framework for future research in our understanding of brain evolution and novel behavioural traits.

## 1. Introduction

The evolution of brain pathways for generation of complex behavioural traits remains an enigmatic and fundamental question in biology. Therefore, examining the proximate and ultimate mechanisms driving changes in brain structure and function provides an exciting opportunity to understand the evolution of complex behavioural traits. In this regard, various hypotheses have been proposed to explain evolution of complex behavioural traits, including increases in brain or brain region size relative to body size, increases in total number of neurons or neuron density, and presence versus absence of particular neural networks that control a specific type of behaviour [1–5]. Some such changes may have occurred with the emergence of the telencephalon during the invertebrate to vertebrate transition, indicating that the central nervous system has been an important target of selection [4,6–8]. However, current empirical evidence for such models and theories are few and wanting.

Another fundamental problem in explaining the evolution of complex behaviours and brain pathways is understanding the contributing cellular and molecular mechanisms. One overall hypothesis is that significant changes in the brain can be generated by novel gene functions owing to gene duplications or expansion of gene regulatory networks [7,9–12]. One of the duplicated genes may then acquire a mutation in coding or regulatory sequences, which enables it to acquire a new function that then undergoes selection, a process known as neofunctionalization [12–15]. Other hypotheses posit that existing genes are modified, including changes in coding sequence, *cis*-regulatory motifs [16,17] or new alternative mRNA splice variants [18–21], a process known as subfunctionalization [12–14]. However, the origin and evolution of such molecular changes in the evolution of the nervous system and behavioural complexity are not well understood.

Here, we review and expand upon an underappreciated theory of evolution of brain complexity, namely by brain region or whole brain pathway duplication from pre-existing brain circuits. We propose hypotheses on cellular and molecular mechanisms for brain region and pathway duplication, including by gene duplication. We believe that such mechanisms may form a cornerstone of evolution of brain and behaviour complexity, which enable adaptations to new environments and social situations.

## 2. Theories on brain region and brain pathway evolution for brain complexity

Comparative neurobiology studies indicate that many primitive features of brain organization have been preserved to varying degrees in extant species [22]. The brain also has evolved in a mosaic pattern, with some regions changing dramatically, while others have remained little changed through the course of evolution [23]. While it is still unclear how brains evolve, past theories posit that brain evolution could be understood by examining how brains develop embryonically and how such development can be modified [22]. It is thought that the early embryonic state of the brain across species represents a more similar and thus ancestral state, and that during development, brain cells, regions and pathways diverge towards lineage- or species-specific states. This is one way in which homologous brain regions can become diverse in adults across species. Based on this view, the vertebrate brain is proposed to consist of three basic divisions, with the spinal cord and brainstem (hindbrain, midbrain and thalamus) having more conserved organization, and the telencephalon more divergent organization [24]. In turn, the telencephalon consists of three major subdivisions, with the pallidum and striatum having more conserved organization and the pallium or cortex a more divergent organization. The pallium is largely layered in mammals, and mostly nuclear in birds, reptiles and other vertebrates, but with divergences among them [24,25].

With these fundamental principles, one can argue that divergences may occur in many forms leading to more behavioural complexity, including: (i) larger brain-to-body size ratios endowing those animals with more advanced abilities [3]; (ii) novel connectivity within a pre-existing brain circuit that enhances that particular circuit's function for complex behaviours [26,27]; and (iii) the de novo presence of a specific brain region or circuit that controls a newly evolved behaviour, as has been proposed for the evolution of brain pathways for vocal learning and spoken language [2,28,29]. It is this latter theory that requires greater explanation.

A long proposed explanation for generating increased cortical complexity is that a single region gradually differentiates into two or more areas [30–35]. This could occur by expansion of an existing region and then selectively partitioning part of the older region to the new function, while the other part maintains the old function [36]. Allman and Kaas also proposed that development of the brain could be altered owing to a gene mutation so that a given area is duplicated [33,37]. The duplication event would modify the function of either one or both of the ancestral and duplicated areas to take on a new function. Duplication itself may modify the selection pressure on both structures, thereby allowing the individual structures to use the once single functional space in a mechanism reminiscent of adaptive radiation [38]. More recently, Feenders *et al.* [39] suggested that whole brain pathways could duplicate, followed by divergence of one of the duplicated copies. This idea was proposed as a mechanism to explain what they called the *Motor Theory of Vocal Learning Origin*, which we review next.

### (a) The motor theory of vocal learning origin and brain pathway duplication

**Figure 1.**
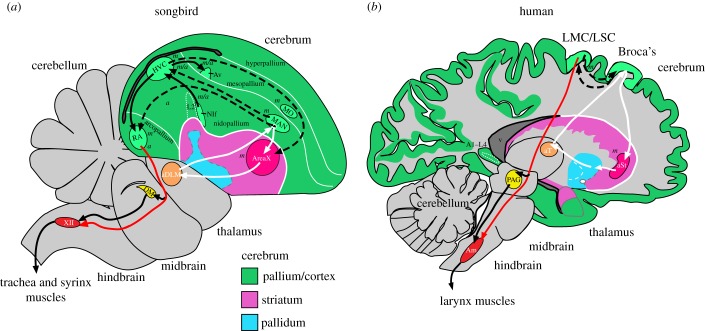
Brain pathways controlling song in songbirds and spoken language in humans. (*a*) Vocal learning song pathway of songbirds. (*b*) Spoken language pathway of humans. Black arrows, posterior vocal motor pathway; white arrows, anterior vocal learning pathway; dashed arrows, connections between the two pathways; red arrows, specialized direct projection from forebrain to brainstem vocal MN in vocal learners. Italicized letters indicate that these regions mainly show motor (*m*), auditory (*a*), equally both motor and auditory (*m*/*a*) neural activity or activity-dependent gene expression in awake animals. Adapted from [2,50]. Not all connections are shown, for simplicity. Some connections in the human brain are proposed based on known connectivity of adjacent brain regions in non-human primates. A1–L4, primary auditory cortex—layer 4; Am, nucleus ambiguous; aSt, anterior striatum; Av, avalanche; aDLM, anterior dorsolateral nucleus of the thalamus; DM, dorsal medial nucleus of the midbrain; HVC, a vocal nucleus (no abbreviation); L2, field L2; LMC, laryngeal motor cortex; LSC, laryngeal somatosensory cortex; NIf, interfacial nucleus of the nidopallium; MAN, magnocellular nucleus of the anterior nidopallium; MN, motor neurons; MO, oval nucleus of the anterior mesopallium; PAG, peri-aqueductal gray; RA, robust nucleus of the arcopallium; v, ventricle space.

Vocal learning, a critical component of spoken language acquisition, is the ability to modify acoustic and/or syntactic features of sounds produced, including vocal imitation and improvization. Vocal learning is a rare trait, so far discovered in five distantly related groups of mammals (humans, bats, elephants, cetaceans (dolphins and whales) and pinnipeds (seals and sea lions)) and three distantly related groups of birds (parrots, songbirds and hummingbirds) [1,40–42]. In the past few decades, significant advances have been made in guiding our understanding of the evolution and mechanisms of brain pathways for vocal learning in birds and humans [2,40,42–49] (figure 1).

The independently evolved lineages of vocal learning birds and humans share distinct forebrain pathways that control the acquisition and production of learned vocalizations [2]. Within these pathways, all three avian lineages contain seven cerebral (telencephalic) vocal nuclei and several thalamic nuclei. These nuclei, best characterized in songbirds and parrots, are distributed between two subpathways (figure 1*a*): (i) the vocal production, or posterior, pathway that influences the production of learned song—which includes an arcopallium nucleus (songbird RA (robust nucleus of the arcopallium), parrot AAC (central nucleus of the anterior arcopallium), hummingbird VA (vocal nucleus of the arcopallium)), analogous to the laryngeal motor cortex (LMC) in humans (figure 1*b*) that makes a specialized direct projection to brainstem vocal motor neurons (MN), which in turn controls the vocal organs, the syrinx (birds) and larynx (humans); and (ii) the vocal learning, or anterior, pathway that is primarily responsible for vocal imitation and plasticity, which forms a pallial–basal ganglia–thalamic loop, analogous to such loops in the mammalian brain that presumably include Broca's speech area in humans. The song and speech regions in both these pathways are embedded in or adjacent to non-vocal motor brain areas [39,50]. These non-vocal motor regions are present in other vertebrate species examined thus far, and are thought to be involved in the production and learning of non-vocal motor behaviours. Based on these findings, Feenders *et al.* [39] proposed a *motor theory of vocal learning origin*, which stated that ‘cerebral brain pathways for vocal learning in distantly related animals evolved independently as specializations of a pre-existing motor system inherited from their common ancestor’ ([39], p.1). This was a more general theory of the *motor theory of language origin* [51], but with specific brain regions identified and a proposed mechanism. The motor theory of vocal learning origin suggested that the last common ancestor of birds and mammals had a motor forebrain pathway, including a motor cortex or pallium region. This is because although the motor pallial domain in mammals consists of six layers of cells (layered) and nuclear subdivisions in birds and reptiles (clustered), they function similarly and developed from the same embryonic primordium. This is supported by results obtained from activity-dependent gene expression and differential gene expression experiments, which show that the avian pallium has a functional columnar organization similar to the mammalian pallial domain [39,52–54]. Further, the mammalian non-vocal motor descending pathway and the pre-motor pathway share similar connectivity patterns in avian posterior and anterior motor pathways, respectively, suggesting the presence of a pre-existing motor system shared by both groups and their most recent ancestor [1,39,55,56].

The proposed mechanism of evolution of vocal learning pathways was by brain pathway duplication [39]. In this regard, it was hypothesized that parallel forebrain motor learning pathways with auditory, somatosensory or other sensory input, normally replicate multiple times during embryonic development and connect to different brainstem and spinal cord neurons to control different muscle groups. In vocal learners, this forebrain pathway is hypothesized to replicate one more time and then connect to the brainstem circuits that control vocalizations and respiration. Then the new vocal learning pathway would diverge to form novel connections and functions relative to the adjacent non-vocal motor pathways. Under this duplication hypothesis, the vocal learning pathways share a deep homology with the surrounding motor pathways, but convergence in the independent lineages of vocal learners.

Several alternative hypotheses have been proposed for evolution of vocal learning pathways, including that the pathways in humans and song-learning birds originated out of either a pre-existing auditory pathway [57,58], a non-motor cognitive region [59,60], a combined auditory–motor pathway [61], or completely de novo [62]. In support of an auditory origin hypothesis, the songbird posterior vocal motor pathway is also partly adjacent to the auditory pathway and has some parallel connections with the descending auditory system [58]. However, such an anatomical position is not present in hummingbirds, parrots, or humans [1,2]. With the exception of the completely de novo hypothesis, even if the vocal learning pathway arose from a non-motor pathway, the hypothesis of pathway duplication could still apply.

If the duplication hypothesis were true, then one would expect to find most genes expressed in vocal learning pathways to be similar to the pathway from which they were duplicated. Further, one would expect to find divergent molecular changes in neural connectivity genes associated with the unique connections found in vocal learning pathways. These ideas were recently tested in a high-throughput gene expression study using a novel computational approach that determines homologous and convergent specialized anatomical gene expression profiles from thousands of samples and genes from multiple species [50]. Using comparative microarray gene expression profiling of approximately 3000–7000 genes in vocal learning and vocal non-learning avian and primate species, Pfenning *et al.* [50] found that the song and speech brain pathway regions of vocal learning birds and humans have gene expression profiles that more closely match motor and premotor cortex and striatal pathway regions adjacent to them than they do to auditory, somatosensory or other brain regions (figure 1). These results corroborated some earlier single gene expression, developmental, functional, and connectivity studies [24,52,63–67]. Combined, the findings support the idea that the similarities are owing to homology and not convergence. Further, Pfenning *et al.* [50] found divergent changes in expression of genes that control neural connectivity in the avian song and the human speech regions from the surrounding motor areas, but that were convergent among the vocal learning birds and humans. There were also convergent changes in some genes involved in neural development, neuroprotection and synaptic communication functions. The brain expression characterization of these genes [50,68] led to the discovery of a further apparent duplication in the parrot brain [56], as described next.

### (b) Parrots contain a song system within a song system

Parrots surpass other vocal learning avian species in their ability to imitate human speech and also rival non-human primates in their display of advanced cognitive skills and ability for tool use [2,69–74]. From 1981 until recently in 2015 (approx. 34 years), the neural pathways for vocal learning had been studied in only one parrot species, the budgerigar (*Melopsittacus undulatus*) [55,75–79]. From these studies, it was apparent that the budgerigar song system shows some differences from the songbird and hummingbird song systems [55,75,76,79,80]. The posterior song system of songbirds and parrots (and presumably hummingbirds) receives auditory input from the posterior auditory pathway (e.g. auditory Field L), but in parrots it receives additional auditory input from a small part of the nucleus basorostralis (B), the remainder of which is somatosensory [81]. Neural tracing and singing-regulated immediate early gene studies revealed some differences in connectivity and position or shape of song nuclei in parrots, but no clear differences were noted in the presence or absence of song system structures [55,75,76,78–80].

**Figure 2.**
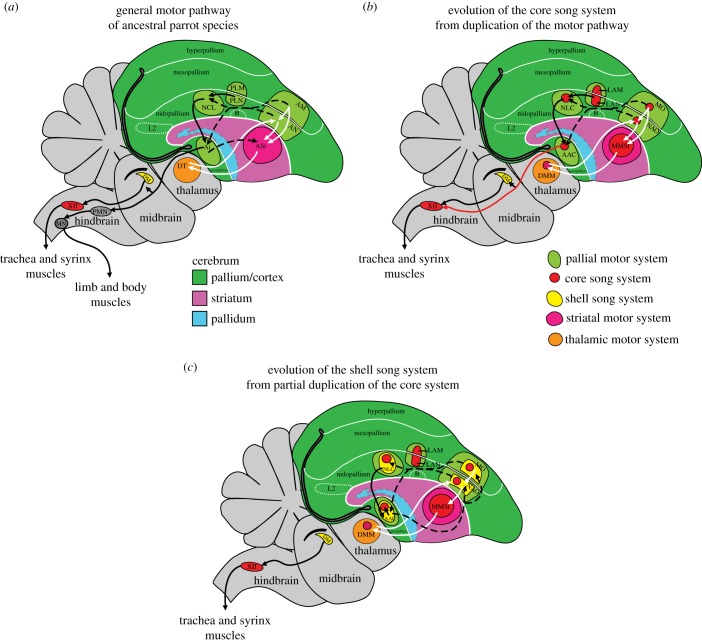
Hypothesis of evolution of song system in parrots owing to sequential pathway duplications. (*a*) The parrot ancestral motor pathway (light green) with the posterior motor connections (in black arrows) and the anterior motor connections (in white arrows). (*b*) The parrot core song system (red), proposed to have evolved out of the pre-existing motor pathway through duplication. (*c*) The parrot shell song system (yellow), proposed to have evolved out of a partial duplication of the core song system. Black arrows, posterior vocal motor pathway; white arrows, anterior vocal motor pathway; dashed arrows, connections between the two pathways; red arrow, specialized direct projection from forebrain to brainstem vocal MN in vocal learners. Connectivity based on summaries in [39,55,56,76,83,84]. See fig. 20 from Chakraborty *et al.* [56] for additional connections of the core and shell song pathways. Not all connections are shown for simplicity, including reciprocal connections and additional thalamic projections. AAC, central nucleus of the anterior arcopallium; Ai, intermediate arcopallium; AM, anterior mesopallium; AN, anterior nidopallium; ASt, anterior striatum; B, basorostralis; DM, dorsal medial nucleus of the midbrain; DMM, magnocellular nucleus of the dorsomedial thalamus; DT, dorsal thalamus; L2, L4, auditory areas; NAO, oval nucleus of the anterior nidopallium; NCL, nidopallium caudal lateral; NLC, central nucleus of the lateral nidopallium; PMN, premotor neurons; LAN, lateral nucleus of the anterior nidopallium; LAM, lateral nucleus of the anterior mesopallium; MMSt, magnocellular nucleus of the medial striatum; MO, oval nucleus of the anterior mesopallium; PLM, posterior lateral mesopallium; PLN, posterior lateral nidopallium; XII, 12th motor nucleus.

Recently, partly based on the high-throughput gene expression [50,68] and other findings, we led a study [56] characterizing gene expression profiles that are specialized in avian and/or human song/speech vocal learning circuits (e.g. *PVALB*, *SLIT1*, *FOXP1*, *NR2A*, *GLUR1*, *NADPH-d*, *AR*, *mENK*, *TH*, *CGRP-LI*) to understand the organization of the song system in diverse groups of parrots representing all the three superfamilies, Strigopoidea, Cacatuoidea and Psittacoidea [82]. We found that the parrot pallial (cortical) song nuclei had core regions that differed in gene expression from surrounding shell regions, and both in turn differed from the surrounding cortical motor areas (figure 2) [56]. Surprisingly, a subset of the genes (including *PVALB*) had moderate specialized expression in the immediate surrounding non-vocal motor areas. Both the core and shell song regions were functionally active in the production of learned vocalizations, as revealed by vocalizing-driven immediate early gene expression (*EGR-1*, *C-FOS* and *DUSP-1*), whereas the surrounding brain regions were active in production of non-singing motor behaviour, as revealed by rhythmic controlled hopping-driven gene expression [39,56,79,85,86].

The connectivity of the core and shell systems were similar to each other, but with some significant differences. One fundamental difference was that the core system made the rare direct projection to brainstem vocal MN (via the AAC core nucleus), whereas the parallel shell song system made mostly intra-cortical projections (via AAC shell; figure 2*b*,*c*). The direct projection to the brainstem vocal MN is considered critical for the evolution of vocal learning and spoken language, as it is either absent or very sparse in vocal non-learners [2,42,87–92]. There were sparse connections between the parrot cores and shells within and among each song nucleus.

The presence of these song nuclei in the kea, the most distantly related to the other extant parrot species [82,93], indicates that parrots evolved the core and shell song systems over 29 Ma before the kea split from the other parrot lineages. The kea shell system, however, was less well differentiated in terms of its gene expression specializations. There were also large species differences in relative sizes of the core and shell regions, where the shell had a significant log-linear relationship with their brain section size, but the core did not. This meant that shell regions were relatively larger in species with bigger brains such as the gold and blue macaw, and the African Grey and Amazon parrots that are considered to have more advanced communication and cognitive capacities.

The fact that the shell system AAC nucleus does not have the direct projection to the vocal MN (which is restricted to the core region of AAC), but may be correlated with more complex vocal learning behaviour, indicates that such direct projections may not be the only means to increase learned motor behavioural complexity over innate motor behaviours. We speculate that it is possible that the direct projection may not be required for the ability to imitate complex vocalizations, but strictly for the production of those learned vocalizations. Clearly, further studies will be required to explicitly test this hypothesis in parrots.

The gene expression specializations and neural connectivity of the parrot core song nuclei were most similar to the song nuclei of songbirds and hummingbirds, whereas the shells were unique to parrots. The shell specializations appear to be restricted to the cortical parrot song nuclei, as no shells have yet been found for the striatal or thalamic song nuclei. The songbird and hummingbird species examined to date do not have parallel vocal motor shell regions for any of their nuclei.

These findings indicate that the core and shell are two parallel, partially independent systems, performing similar and some unique functions for vocalizations. They support a partial brain pathway duplication hypothesis of brain evolution. In particular, we suggest that the core song system evolved convergently in parrots, songbirds and hummingbirds as a duplication event in each lineage from the surrounding motor areas (figure 2*a*,*b*). Thereafter, the parrot core cortical song nuclei underwent a further partial duplication event to evolve the shell song system (figure 2*c*). The shell song system went on to evolve specializations that allow more complex vocal communication abilities in parrots compared to other avian vocal learners. This dual system evolved early in the parrot lineage, and has lasted and expanded for millions of years in different species. In addition, changes in the regulation of some genes that may allow greater vocal–motor–auditory integration in vocal learning systems could have influenced changes in the surrounding motor areas to allow greater auditory–motor entrainment and synchronizing of body movements to the rhythm of music for dance in parrots [94–96].

It would be exciting to determine if similar duplications of brain pathways have occurred in humans, not only in the speech pathways but also for other advanced motor movements such as dancing [97–99]. In the human brain, areas 44 and 45 constitute Broca's area, the ventrolatreal frontal region critical for spoken language acquisition and production [100]. Petrides *et al.* [101,102] showed that there is a comparable area 44-like region involved in orofacial musculature functions in macaque monkeys. What is still unknown is whether area 44 in the human and the macaque brain share common ancestry since there is lack of an outgroup comparison so far. It is tempting to speculate that Broca's area could constitute one or more duplications of a more ancestral area 44, with divergent specializations for learned vocalizations and thus spoken language.

## 3. Some alternative hypotheses to the motor theory and brain pathway duplications

Expanding upon the alternative hypotheses, it is possible that the core and shell systems in parrots arose simultaneously, in which case the shell would not be a duplication of the core. However, for this alternative, it would be difficult to explain why the parrot core pathway is more similar to the songbird and hummingbird song systems, than an apparently evolutionary older state (i.e. appearing first). The shell song system of parrots does not appear to be a functionally differentiated region of the core system, since the core system still exists in them and other vocal learners.

A second alternative is that the shell is an independent duplication of the adjacent motor pathway, as the shell has a more similar gene expression profile to the adjacent motor pathway than the core. However, if this were the case, it would be hard to explain how evolution of the shell is more independent of the core, considering that the shell song nuclei surround the cores and are not located elsewhere in the motor pathway and that both core and shell are interconnected without notable major connections to the surrounding motor areas.

A third alternative is that the parrot shell and core song nuclei, as well as the songbird, hummingbird and human song/speech region analogues, are all specialized transformations of an existing motor pathway, i.e. subfunctionalizations, rather than duplications from it, i.e. neofunctionalizations. This would mean that the vocal learning species lost non-vocal motor learning pathway neurons to gain vocal motor pathway neurons. However, there is as yet no evidence for loss of non-vocal motor or other functions in vocal learning species, but rather gain of functions even beyond vocal learning (such as learning how to dance) [94,96,97,99] and increases in relative brain-to-body sizes [103].

A fourth alternative is that vocal learning pathway neurons migrate into their adult locations from developing non-adjacent and even non-motor brain areas, and then by adjacent association they adopt some of the motor learning pathway phenotypes. Although this alternative is theoretically possible, one would expect that the vocal learning pathway cells would have some vestigial properties of their non-motor origin. Thus far, the evidence has not revealed such an alternative origin, although only in songbirds does the HVC (a letter-based name) motor pathway nucleus share some secondary profiles in gene expression with the human secondary auditory cortex [50].

A bigger challenge to the motor theory and duplication hypotheses might at first glance be derived from a recently proposed ‘continuum hypothesis' of vocal learning. Based on findings that mice have a rudimentary forebrain circuit involved in modulating vocalizations but with a very sparse direct projection to brainstem vocal MN, buried within a motor region that also controls other behaviours, and that mice and non-human primates have at least a very limited ability to modify their vocalizations based on auditory experience, Arriaga & Jarvis [104] and Petkov & Jarvis [2] proposed a continuum hypothesis of vocal learning. In this hypothesis, vocal learning is considered to range from complex (e.g. humans and parrots), moderate (some songbirds), to limited or none (mice and non-human primates) [105–108]. In this model, there would be a rudimentary pre-existing forebrain vocal pathway within the vertebrate motor learning pathway, but in the more complex vocal learners these vocal pathway neurons independently expanded and segregated out of the motor learning pathway. However, it is also possible that initially the motor learning pathway duplicates within the non-vocal motor learning circuits to form a limited vocal learning circuit, which subsequently evolves enhanced functions and moves outside of to become adjacent to the motor learning circuit.

Resolving these hypotheses will require more comparative research. The nuances for limited vocal plasticity and an associated neural circuit in mice and non-human primates are also still an ongoing debate that requires further investigation [104–111]. Thus far, of all the hypotheses proposed, we believe the existing data most support the motor origin and duplication hypothesis for vocal learning pathways.

## 4. Other examples of duplicated morphological structures and structural subdivisions in the evolution of functional complexity

If brain pathway evolution by duplication were possible for vocal learning circuits, then it could be one broad mechanism of brain evolution. The presence of the well-known parallel cortical–basal ganglia–thalamic–cortical loops through the anterior forebrain of mammals and birds is consistent with such an idea. These parallel loops could be replicates of a basic motor learning pathway design. Since all of the cortex is connected with all of the basal ganglia and thalamus [112], when a cortex region is duplicated, one would expect to see a concomitant duplication in the connected basal ganglia and thalamic regions [22,33]. However, our finding of only cortical shell song nuclei duplications thus far in parrots indicates that it could be possible that the duplicated cortical regions co-innervate the non-duplicated striatal and thalamic regions (figure 2*c*). Such flexibility would allow for greater diversity in neural circuit evolution balanced with the constraint of limited cranial space to accommodate increases in brain size owing to duplications.

Studies of non-human primate motor cortex are consistent with the idea of duplicated brain components. For example, studies using retrograde transneuronal transport of the rabies virus from single muscles of rhesus macaque monkeys to identify layer 5 cortico-motoneuronal (CM) cells in the primary motor cortex (M1) have shown that the M1 region has two subdivisions [113]. The rostral subdivision of macaque M1 has been proposed to be an ‘older’ region as it contains fewer CM cells that make indirect projections to spinal cord MN and is present in most mammals requiring the indirect use of the spinal cord to influence motor output. The caudal subdivision of macaque M1 is proposed to be ‘newer’ as it contains the more rarely found CM cells that make direct projections to MN in the spinal cord and brainstem, including controlling shoulder, elbow and finger muscles to produce highly skilled motor actions. Based on these and other findings, it is generally proposed that the direct CM system of M1 is a recently evolved brain structure that conferred evolutionarily novel functions in the motor system in primates, including independent voluntary control of finger movements, which are more advanced in primates compared with non-primates [114–120]. Assuming that the evidence continues to support differences between species, one hypothesis, like the one we propose for vocal learning pathways, is that the newer caudal M1 region is a duplication of the older rostral M1 region, but with a divergent connection of the CM cells from cortical layer 5.

Examples of morphological duplications or subdivisions to enhance complexity also exist outside of the nervous system. Many animals, such as annelids, have repeated parallel body segments or specialized limb types among species, where the replicated parts are thought to be owing to a repeating developmental genomic programme [121,122]. A striking example of independently evolved morphological duplications is the diversification of the adductor mandibular muscles in teleost fish jaws, which have independently subdivided several times during tetraodontiform evolution [123–125]. Most of these divisions have been incomplete, which suggests that some parts were subfunctionalized instead of duplicated. The duplicated adductor mandibulae muscles continue to maintain similar morphological characteristics, but with increased morphological complexity associated with their functional complexity resulting in finer motor control for feeding [125]. Overall, structural duplications have been proposed to be one mechanism that allows for morphological decoupling [126]. Making structures, such as brain circuits, functionally independent of one another may provide increased complexity and opportunity for modification and diversification [126–128]. In this regard, brain evolution by brain region duplication, brain pathway duplication or structure subdivision may follow a general mechanism of morphological evolution to enhance functional complexity. Testing these hypotheses will be best informed by deciphering the cellular and molecular mechanisms for the development of additional, parallel circuits in the brain.

## 5. Proposed cellular and molecular mechanisms for evolution of brain pathway duplications

During development, neural stem cell/progenitor cells that give rise to forebrain circuits derive mainly from stem cells in the ventricular zone [129–131]. The daughter cells travel to their positions either by radial migration perpendicularly away from the ventricle (such as excitatory neurons within layers of the mammalian cortex), and/or by tangential migration parallel to the ventricle (such as inhibitory neurons that migrate from the basal ganglia into the cortex) [132–134]. Their local brain region identity is thought to be controlled by patterning transcription factors, such as the homeobox (*Hox*) genes [135,136]. Once daughter cells reach their target location in the brain, they find their connecting partners in a process that requires cell adhesion and axon guidance genes [137,138]. Given these principles, we propose that one possible mechanism for the evolution and development of duplicated segmented brain circuits is that there is a set of transcription factors that not only control the position of such circuits, but also the number of replicates of that circuit. A genetic change in such transcription factors could result in a new parallel circuit, such as that for vocal learning. Thereafter, changes in axon guidance genes in the new circuit would control divergence in connectivity relative to the older circuit. This begs the question of what kind of genetic change would this be?

We propose that gene duplication could be one such mechanism. Gene duplications have been found to influence the development and function of many organs and tissues, including brains, eyes and wings [11,139–147]. As proposed originally by Ohno [13,14], many consider gene duplication to be one of the most important factors in evolution, including neofunctionalization, subfunctionalization and evolutionary innovations. Gene duplication allows the old gene copy to maintain its function and the new copy to evolve new functions. Even theories on gene evolution through gene duplication have influenced the theories on brain evolution by morphological duplication [139,141]. The concept of neofunctionalization of genes [14] and subfunctionalization of genes [148] match those proposed for structural duplications [128,149,150].

One of the most well-studied and significant examples of duplicated genes controlling duplicated, repeated or segmented morphological structures are the *Hox* genes. These are transcription factors that control the anterior–posterior body plan axis and are situated in the genome in the same order as the body plan they control [136,151,152]. They are duplicated to different degrees in different animal lineages, with greater complexity and more anatomical segments correlated with more duplications [153]. Many invertebrates and *Amphioxus* possess one *Hox* gene cluster, whereas the remaining vertebrates have four *Hox* gene clusters, in part owing to two whole-genome duplication events that occurred early in vertebrate evolution [151,154–158]. Within the brain, the *Hox* genes and the greater *Hox* gene transcription factor superfamily (including *OTX*, *EMX*, *DMBX*, *GBX* and *EN*) are involved in brain division and subdivision segmentation [135]. They do so by controlling regional neuronal identity, stem cell progenitors, cell migration and cell death [159,160].

We propose that one possible mechanism for brain pathway duplication could be a local duplication of *Hox* superfamily genes in the genome segments that control forebrain development. One prediction of this hypothesis is that one should find such genes uniquely duplicated in vocal learning species that control brain development. Recently, based on comparative genomic analyses across the bird family tree, unique gene duplications were found in the songbird lineage and some of these genes had enriched or nearly exclusive expression in the song learning nuclei [161]. It remains to be determined, however, if any of these transcription factors belong to the *Hox* gene family.

We caution that we are not suggesting a one-to-one relationship of gene duplication with morphological duplication. There are many examples of gene duplications resulting in modifications of existing structures and functions. An example relevant to the topic of vocal learning and cognition is the *Slit-Robo GTPase 2* gene (*SRGAP2*), which has undergone two partial duplications (*SRGAP2B* and *SRGAP2C*) uniquely in humans relative to other mammals [26,27,162]. The duplicated copies act as competitive inhibitors to slow cortical dendritic development of already existing brain pathways, which in turn allow greater neural plasticity into adulthood. *SRGAP2* modulates activity of the ROBO axon guidance receptors, which are in turn activated by the SLIT family of protein ligands to modulate axonal/dendritic migration and branching in various brain regions [163–167]. Intriguingly, the *SLIT1* ligand is uniquely downregulated in the song production nucleus RA analogue of vocal learning birds (songbird RA, parrot AAC and hummingbird VA) [56,68] and the analogous human LMC [50], which would mean that there could be a synergistic effect of the duplicated *SRGAP2* GTPase and lower *SLIT1* levels in the duplicated vocal motor pathways in humans. Another recent example of partial duplication includes another GTPase, the *ARHGAP11B* gene, which arose from *ARHGAP11A* in humans after separation from the chimpanzee lineage [168]. The duplicated copy of the *ARHGAP11A* gene causes cortical area expansion, and this expansion causes folding, which we surmise could be owing to the duplication.

Advances in genetic technologies have also allowed scientists to test some hypotheses on duplicating or eliminating neural structures genetically. For example, ectopic visual responsive eyes were induced in *Drosophila* with the addition of an extra copy of one transcription factor, *Pax6*, expressed during development in another part of the body [169]. Another study showed that electroporating an extra copy of the fibroblast growth factor 8 (*FGF8*) gene locally in the posterior cortical primordium of mouse embryos causes a partial duplication of the primary somatosensory cortex, with concomitant input from the thalamus to its layer 4 cortical cells, as shown by the presence of ectopic somatosensory barrel fields [170]. In vertebrates, the expression of the *Hox1a* gene marks the earliest stages of regionalization of the developing hindbrain. Mice mutant for the *Hoxa1* gene lack the developing rhombomere 2 (r2) brain region, but the r2 neurons escape apoptosis and develop within r3 and r4, to still incorporate into appropriate circuits to drive the rhythm of breathing [171]. This suggests to us that *Hox1a* is needed for development of a separate, repeated rhombomere region, but that other factors are sufficient to develop the associated circuit within another circuit. An example of loss of gene function and functional redundancy leading to duplication of structure is the Mauthner (M) cells, a pair of reticulospinal neurons that control escape behaviour in zebra fishes [139,172–174]. During the escape response, if the threatening stimulus arrives from the left side, the left M cell fires, and its action potential travels to the right side so that the fish swerves to the right side owing to the contraction of the muscles on the right side to avoid the threat. Mutation of the *notch1a/deadly seven* (*des*) in zebra fish results in the development of extra M cells in r4 [175]. All extra copies of the M cells are responsive to the escape stimuli, suggesting that when duplication of the cells takes place, they receive the appropriate sensory information and respond in a normal way indicating adaptive plasticity of the escape-response circuit.

Other plausible hypotheses of molecular mechanisms that could lead to brain pathway duplication include: (i) changes in splice variants of a gene [176], which we propose could switch on and off at different developmental times to control the generation of parallel circuits; (ii) changes in the *cis*-regulatory elements of genes, which we propose again would change the reiterative use of a gene network in parallel developing circuits; and (iii) loss of function in a gene that may normally inhibit development of some circuits.

Overall, the various hypotheses may be tested with recent advances in genomics, transcriptomics and gene manipulations, using complete genome sequences from multiple species with and without the brain pathways of interest [93]. Until then, the existing evidence supports the possibility that *brain pathway evolution through brain pathway duplication* could be one mechanism to generate higher-order complexity in highly evolved animals.

## 6. Conclusion

In this review, we discussed new evidence from studies in birds, primates and other species that suggests that brain pathways for a novel convergent trait, vocal learning, possibly evolved by duplication from adjacent motor learning pathways. The continuum hypothesis of a pre-existing vocal learning pathway that was independently enhanced in vocal learners is an alternative, but could be compatible with the duplication hypothesis if the duplication occurs within an existing pathway, as seen with *Hox1a* r2 manipulations. Whether by duplication or enhancement, the pathways have diverged from their adjacent brain regions by specializations of genes involved in neural connectivity. These divergences may have been heavily selected upon for immediate and substantial phenotypic benefits. Despite these divergences, the vocal learning circuits share many properties with the adjacent motor pathways. The findings of the parrot core and shell song system lead us to wonder if humans evolved consecutive or simultaneous multiple duplications of a vocal learning pathway leading to more advanced spoken language abilities. Moreover, findings from studies outside of the vocal learning systems indicate that brain region or pathway duplication could be a general mechanism of brain evolution.

Answers to these questions can now be determined through comparative neurobiology and comparative genomics research. With the recent availability of genomes across the avian [93,177,178] and eventually primate [179,180] family trees, it becomes possible to discover candidate genes. They can then be studied with advanced technologies, such as transcriptomics and genome editing tools, including CRISPR-Cas9, RNAi, TALENs, Cre-Lox systems and more. The theoretical framework presented here will help guide use of these technologies.
